# Time-course whole blood transcriptome profiling provides new insights into *Microtus fortis* natural resistance mechanism to *Schistosoma japonicum*

**DOI:** 10.1016/j.heliyon.2024.e38067

**Published:** 2024-09-26

**Authors:** Nouhoum Dibo, Zhijun Zhou, Xianshu Liu, Zhuolin Li, Shukun Zhong, Yan Liu, Juan Duan, Meng Xia, Zhenrong Ma, Xiang Wu, Shuaiqin Huang

**Affiliations:** a Department of Medical Parasitology, Xiangya School of Basic Medicine, Central South University, Changsha, 410013, Hunan Province, China; b Department of Laboratory Animals, Hunan Key Laboratory of Animal Models for Human Diseases, Central South University, Changsha, 410013, Hunan, China; c Hengyang Medical College, University of South China, Hengyang, 421001, Hunan Province, China; d The Third People's Hospital of Hunan Province, Yueyang, 414000, Hunan, China; e National Key Clinic on Schistosomiasis, Yueyang, 414000, Hunan, China; f Schistosomiasis Control Institute of Hunan Province, Yueyang, 414000, Hunan, China

**Keywords:** *Microtus fortis*, Time-course transcriptome profiling, Whole blood, Natural resistance, *Schistosoma japonicum*

## Abstract

*Microtus fortis* is known as a non-susceptible animal host of *S. japonicum*. A better understanding of this animal immune defense mechanism during the early stage of infection may offer an alternative route for vaccine development or therapy. Here, we analyzed the whole blood transcriptome of *M. fortis* using next-generation sequencing (NGS) to identify immune genes of biological relevance that might be involved in the mechanism of its resistance. The blood samples were collected from uninfected animals (control group) and infected animals at different time points (3, 7, 10 and 14 days post-infection). We identified 5310 sequences as unigenes and successfully annotated 4636 of them. The immune response was more intense at 10 dpi. The upregulated genes at this time point were mainly activated in the TNF and NF-kappa B signaling pathways, Th1, Th2and Th17 cell differentiation as well as cytokine-cytokine receptor interaction. Based on the differentially expressed genes analysis, we report that the *IF27L2B*, *RETN*, *PGRP*, *IFI35*, *TYROBP*, *S100A8*, *S100A11*, *CD162*, *CD88*, *CYBA*, and *LBP* could play important roles in the mechanism of *M. fortis* resistance.

## Highlights

• The whole blood transcriptomic is suitable to elucidate the mechanism of *M. fortis* resistance.
• IF27L2B RETN, IFI35, TYROBP, CD162, S100A11and CD88 could play important role in the mechanism of *M. fortis* resistance.
• *S. japonicum* induces an inflammatory response during the early stage of infection *in M. fortis*.

## 1 Introduction

Schistosomiasis is a parasitic disease infecting approximately 250 million people in tropical and subtropical regions [1]. Three main Schistosoma species can affect human: *S. japonicum*, *S. mansoni* and *S. haematobium*. *S. japonicum* is a zoonotic and multi-host pathogen that can infect more than 46 mammal species [2]. Infection occurs when the larval form of the parasite penetrates the skin during contact with infested water [3]. To date, praziquantel (PZQ) is the most available drug used to treat schistosomiasis [4]. However, PZQ cannot prevent reinfection, warranting the need to find alternative drugs or vaccines that can overcome this challenge [5]. Great effort has been made in vaccine research, but there is no commercialized vaccine for human use currently [6].

*M. fortis* is a rodent distributed in China, Mongolia, North Korea and Russia [7,8]. The life cycle of *M. fortis* is divided into four stages: infancy stage (1–10 days old), juvenile stage (11–20 days old), subadult stage (21–50 days old), and adult stage (over 51 days) [9]. In China, they are mainly distributed in the Dongting lake wetland [8]. However, when the water level rises, *M. fortis* migrate into the surrounding areas [10]. The Dongting lake is one of the most important transmission zones of *S. japonicum* to humans and animals in China (F.-Y [11]). Despite the fact that the *S. japonicum* is highly prevalent in these areas, no mature worm or egg was identified in *M. fortis* during the epidemiological surveys [12]. It has been reported that *S. japonicum* can migrate from the skin to the liver after initial penetration but die before reaching maturity in *M. fortis* [2,13]. Therefore, *M. fortis* is recognized as a non-susceptible host of *S. japonicum*.

The elucidation of the immune defense mechanism of the non-permissive hosts may offer an alternative route for human vaccine development [14]. Comparative studies of *M. fortis* and mice (highly susceptible host) immune response have revealed that the intense immune response in *M. fortis* occurs during the early stage of infection [39]. In contrast, no significant immune response was observed in mice during the early stage of infection [13](H [11]). In addition, a recent study demonstrated that cell macrophages of *M. fortis* can adhere to the invading schistosomes and destroy them by trogocytosis during the early stage of infection [15]. Therefore, the early stage of infection is essential to understand the immune response directed against the invading schistosomula in *M. fortis*.

The lack of *M. fortis* commercialized antibody makes it difficult to study the resistance mechanism of this animal using flow cytometry and Western blot. Hu et al. demonstrated that transcriptome sequencing can be used to study the molecular mechanism of *M. fortis* resistance [16]. Because the invading schistosomula migrates and die in the liver of this host, the transcriptome of the liver has been analyzed to investigate the mechanism of *M. fortis* resistance [16](H. [11,17]). However, the transcriptome changes in the whole blood during the early stage of infection have not been investigated. As a connective tissue, blood plays an important role in the functioning of the immune system. The transcriptome profile of the whole blood can provide critical information on the immune networks that operate throughout the entire body of the host [18]. In this study, we hypothesized that the whole blood transcriptome analysis could allow the identification of potential candidate genes associated with the mechanism of *M. fortis* resistance. Therefore we investigated the transcriptome changes in the whole blood during the early stage of infection using high-throughput Illumina sequencing.

## 2 Methods and materials

### 2.1 Animal and parasite materials

*Oncomelania hupensis* snails infected with the *S. japonicum* miracidia were fed on a cellulose paper until further use. Six to eight weeks old *M. fortis* were provided by the animal laboratory center of our school. The animals used in this study were laboratory animals and inbred strains. The animal experiments were approved by the ethics committee of Central South University under license number 2021-KT25.

### 2.2 *Microtus fortis* infection and blood sample collection

**Fig. 1:**
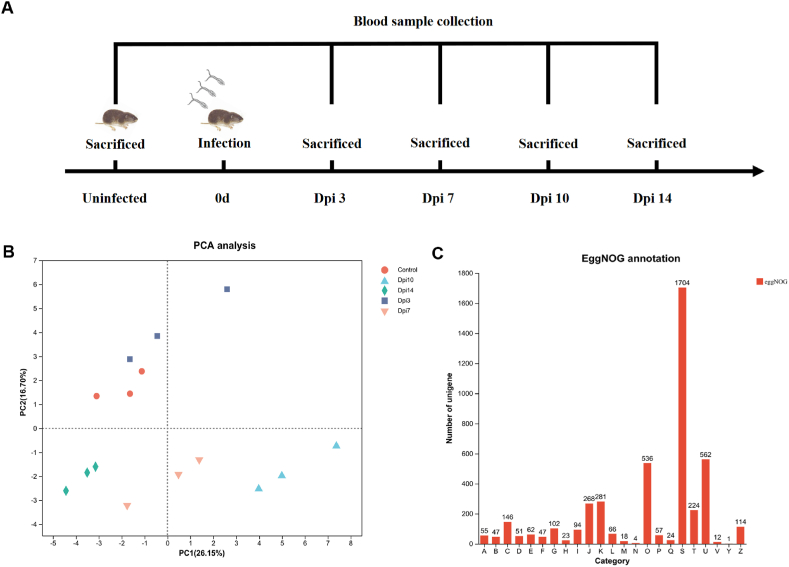
**Sample collection diagram and pre-analysis of the RNA-seq data**. (A) Animal infection and sample collection time points. The blood samples were collected from the control group and infected group at different time points post infection (Dpi3, Dpi7, Dpi10 and Dpi14) to perform RNA-sequencing. (B) PC showing the relationship between samples. Each symbol indicates a group, and there are 3 replicates in each group. The distance of each symbol represents the distance of the sample and the samples with higher similarity are close to each other. (C) Unigenes categorization according to their function in the EggNOG database. A: RNA processing and modification B: Chromatin structure and dynamics C: Energy production and conversion D: Cell cycle control, cell division, chromosome partitioning E: Amino acid transport and metabolism F: Nucleotide transport and metabolism G: Carbohydrate transport and metabolism H: Coenzyme transport and metabolism I: Lipid transport and metabolism J: Translation, ribosomal structure and biogenesis K: Transcription L: Replication, recombination and repair M: Cell wall/membrane/envelope biogenesis N: Cell motility O: Posttranslational modification, protein turnover, chaperones P: Inorganic ion transport and metabolism Q: Secondary metabolites biosynthesis, transport and catabolism S: Function unknown T: Signal transduction mechanisms U: Intracellular trafficking, secretion, and vesicular transport V: Defense mechanisms Y: Nuclear structure Z: Cytoskeleton.

Fifteen *M. fortis* were used in this study: three as a control group and twelve were cutaneous infected with 100 cercariae. Blood samples were collected at 0 day (control group), 3, 7, 10 and 14 days post-infection (DPI) (Fig. 1A). At each time point, three animals were sacrificed and blood samples were collected in a separate EDTA tube to obtain three replicates per time point. The blood samples collected from infected and uninfected animals were sent to Shanghai Majorbio for mRNA sequencing.

### 2.3 cDNA library preparation and illumina sequencing

Trizol reagents were used for the total RNA extraction according to the manufacturer's instruction (Invitrogen) and DNase I (Takara) was used to remove genomic DNA. During the quality control (QC) steps, the NanoDrop-2000 was used for the RNA quantification. The RNA integrity and purity were determined with Agilent 2100 Bioanalyser. The mRNA was captured via Oligo (dT) beads and fragmented into short fragments before cDNA synthesis. The SuperScript double-stranded cDNA synthesis kit (Invitrogen, CA) with random hexamer primers (Illumina) was used to generate cDNA from mRNA. The cDNA libraries were prepared according to the Illumina's library construction protocol and sequenced with the Illumina NovaSeq 6000 sequencer (2 × 150bp read length).

### 2.4 *De novo* assembly and annotation

Fastp was used to control the quality of the raw reads and determine the GC content of the sequences according to the method described by Chen [19]. Trinity was used to generate the transcriptome assembly [20]. High-quality reads obtained after assembly were called transcripts and assembled into unigenes. Optimization and assembly evaluation were carried out using TransRate and Benchmarking Universal Single-Copy Orthologs (BUSCO) [21]. Blastx was used to annotate the unigenes in NCBI-NR, Swiss-Prot, Pfam, COG, GO and KEGG databases.

### 2.5 Differential gene expression screening

The transcripts were quantified by RSEM (http://deweylab.biostat.wisc.edu/rsem/) [22] and the gene expression level was measured according to the transcripts per million reads (TPM) [23]. The R package DESeq2 was used to analyze the DEGs based on the negative binomial distribution (Love et al., 2014). The differences between groups were obtained based on the Padjust and the absolute value of log2 fold change (FC) values (|log2FC| ≥ 1 and adjusted *P* < 0.05). The GO and KEGG enrichment analysis of the DEGs were performed on the Majorbio platform (http://www.majorbio.com) using Fisher's exact test. The GO function and KEGG pathway were considered significantly enriched when the corrected *P-value* was less than 0.05.

### 2.6 Genes clustering

To visualize the expression patterns of the DEGs, we clustered the TPM values of the DEGs using the R package Mfuzz [38]. All genes differentially expressed at least one time point were included in the Mfuzz clustering. The number of clusters was calculated according to the "rule of thumb" and 20 clusters were obtained with 857 DEGs. Since the clustering is performed in Euclidian space, the expression values of the gene were standardized to have a mean of zero, and the highest value was setat 1.5 and the lowest at-1.5.

### 2.7 Quantitative real-time PCR analysis of gene expression

Total RNA was extracted from the whole blood using a Trizol reagent. The RNA was reverse transcribed to generate cDNA using Revert Aid Reverse Transcriptase. Beta-actin (ACTB) was used for internal control. The list of primers used in the study is provided in Supplementary Table 1. SYBR Green using Master Mix on a CFX96 Touch System (BioRad) was used for the RT-PCR. The relative expression was calculated using the 2^**-△△Ct**^ method.

### 2.8 Statistical analysis

GraphPad Prism 9 was used for statistical analyses. The data values were presented as the mean ± standard deviation. T-test was used to compare two groups and the differences were considered statistically significant when the *P-value* was less than 0.05.

## 3 Results

### 3.1 Overview of the assembled sequences

With a total of 15 samples collected at 5 time points (control, 3, 7, 10 and 14 dpi), 131.71 Gb clean data were generated. The clean reads obtained per sample varied between 22781770 and 35747782, and the mapping ratio varied from 14.74 % to 22.78 % (Table 1). After the quality filtration protocols, we obtained 6754 transcripts and 5310 sequences as unigenes (Table 2). Through NR and Swiss-Prot protein database combined with TransDecoder, we identified 4894 protein-coding sequences (CDS). According to their length, the CDS were classified into 10 categories (Supplementary Fig. 1). Some CDS lengths were less than 200 and some CDS lengths were more than 1800. Using Blastx in NCBI, we successfully annotated 4636 unigenes (Table 3). Next, we carried out principal component analysis (PCA) to evaluate the similarity between samples. We found that the control and 3 replicates were clustered closely whereas; the replicates from 7 to 10 dpi were clustered away from the control (Fig. 1B). To classify the unigenes according to their function, we aligned them against the EggNOG database. Among the 4636 unigenes, 4323 were classified into 23 categories. The “function unknown” category contained 1704 unigenes. The largest number of the sequences whose functions are known in the EggNOG database focused on “intracellular trafficking, secretion, and vesicular transport”, “Posttranslational modification, protein turnover, chaperones” and “transcription” (Fig. 1C).

**Table 1:** Reads mapping ratio per sample.

Sample	Clean reads	Mapped reads	Mapping ratio
Mf0_1	29729032	6319258	21.26 %
Mf0_2	31853177	7256927	22.78 %
Mf0_3	31530354	6188561	19.63 %
Mf3_1	30043164	4895464	16.29 %
Mf3_2	33903393	5904420	17.42 %
Mf3_3	22781770	4011403	17.61 %
Mf7_1	23078089	3402219	14.74 %
Mf7_2	35747782	7744059	21.66 %
Mf7_3	28898994	5899810	20.42 %
Mf10_1	29552469	6433598	21.77 %
Mf10_2	32078313	6957107	21.69 %
Mf10_3	28607939	5565813	19.46 %
Mf14_1	29343467	5464034	18.62 %
Mf14_2	27035068	5659662	20.93 %
Mf14_3	29609735	6277354	21.20 %

**Table 2:** Summary of the assembly statistics.

Class	Total number	Total base	Average length	N50	GC %	Fragment mapped%
Transcripts	6754	7943796	1176.16	1589	54.13	23.636
Unigenes	5310	6333414	1192.73	1640	54.19	24.527

**Table 3:** Transcriptome annotation.

Class	GO	KEGG	eggNOG	NR	Swiss-Prot	Pfam	Total_ann
Transcripts	4871	4600	5382	5758	5374	4669	5831
Unigenes	3948	3709	4322	4575	4313	3815	4636

### 3.2 The immune response against the invading schistosome was more intense from 7 to 10 days post-infection

**Fig. 2:**
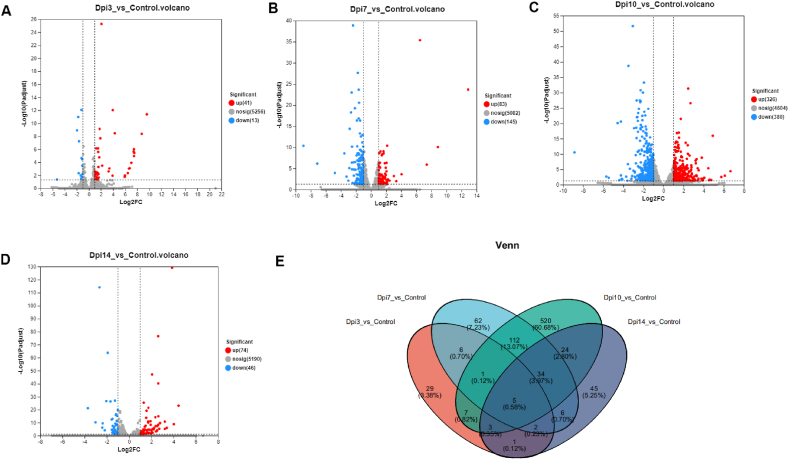
**Differential expressed genes**. (A) Dpi 3 compared to control. The blue color indicates downregulated genes and the red color indicates upregulated genes (B) Dpi 7 compared to control. The blue color indicates downregulated genes and the red color indicates upregulated genes (C) Dpi 10 compared to control. The blue color indicates downregulated genes and the red color indicates upregulated genes (D) Dpi 14 compared to control. Blue color indicates downregulated genes and red color indicates upregulated genes (E) Venn diagram showing the overlap between DEG at different time points. Each circle represents a set of DEGs at one time point. The sum of all numbers within the circle represents the number total of DEG identified at this time point and the cross region of the circle represents the number of common genes between different time points. (For interpretation of the references to color in this figure legend, the reader is referred to the Web version of this article.)

DEG analysis was carried out to identify genes responding to the invading parasite. By comparing each infected group to the control group, we found that 54 unigenes were differentially expressed at 3 dpi (Fig. 2A, Supplementary file1), 228 genes at 7 dpi (Fig. 2B–Supplementary file2), 706 genes at 10 dpi (Fig. 2C–Supplementary file 3), and 120 genes at 14 dpi (Fig. 2D–Supplementary file 4). The DEGs increased from 3i to 10 dpi and decreased at 14 dpi. Five genes overlapped at different points after infection (Fig. 2E). These included the interferon alpha-inducible protein 27-like protein 2B (*IF27L2B*), small nuclear ribonucleoprotein-associated protein N (*SNRPN*), stomatin (*STOM*), carnitine O-acetyltransferase (*CROT*) and serine/threonine-protein phosphatase 2A activator (*PPP2R4*). *IF27L2B* was significantly upregulated from 3 to 14 dpi while *STOM*, *CROT* and *PPP2* were significantly downregulated (supplementary files 1, 2, 3 and 4). In addition, several genes were significantly upregulated from 7 to 14 dpi. These included the resistin-like gamma (*RETN*), peptidoglycan recognition protein (*PGRP*), interferon-induced 35 kDa protein (*IFI35*), TYRO protein tyrosine kinase-binding protein (*TYROBP*), P-selectin glycoprotein ligand 1 (*CD162*), protein S100-A11 (*S100A11*) and C5a anaphylatoxin chemotactic receptor 1 (*C5aR1* also known as *CD88*) (supplementary files 2, 3 and 4). Many other genes such as the S100A8, cytochrome *b*-245 light chain (*CYBA*) and lipopolysaccharide-binding protein (*LBP*) were only significantly upregulated at the two last time points (10 and 14 dpi) (Supplementary files 3 and 4). The DEGs analysis results demonstrated that most DEGs arose from 7 to 10 dpi, and the number of downregulated genes was higher than the number of upregulated genes at these two time points.

### 3.3 Most of the upregulated DEGs were enriched in immune and inflammatory pathways

We performed a separate GO/KEGG enrichment analyses for the upregulated and downregulated unigenes to better understand their function. Compared to the control, no GO categories were significantly enriched at 3 and 7 dpi (Supplementary Tables 2 and 3). Regarding 10 dpi, the upregulated genes were enriched in 103 GO terms mainly involved in leucocyte activation and migration (supplementary file 5) and the downregulated genes in 12 GO terms were mainly related to the ubiquitin system (supplementary file 6). At 14 dpi, no GO categories were enriched for the downregulated genes but the upregulated genes were enriched in 31 GO terms mainly involved in the regulation of immune response (supplementary file 7).

**Fig. 3:**
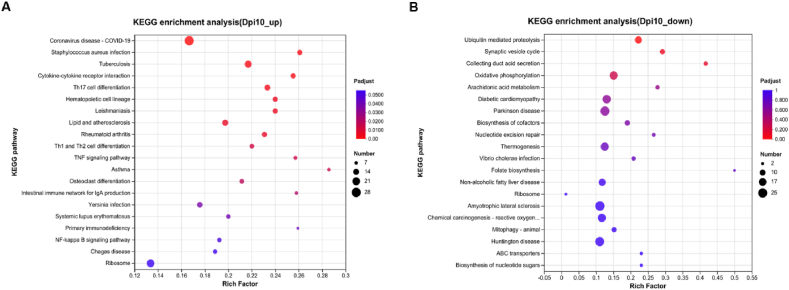
**KEGG enrichment analysis results at 10 dpi**. (A) The most enriched pathways for the upregulated genes. (B) The most enriched pathways for the down-regulated genes.

KEGG analysis revealed that the upregulated genes detected at 3 dpi were enriched in “phototransduction”, “alcoholism”, and “olfactory transduction” (data not shown) but the downregulated genes were not significantly enriched. No significantly enriched KEGG pathway was identified at 7 and 14 dpi. Regarding 10 dpi, the upregulated DEGs were enriched in 18 KEGG pathways including several important pathways such as “TNF and NF-kappa B signaling pathways”, “Th1, Th2and Th17 cell differentiations” and “cytokine-cytokine receptor interaction” (Fig. 3A, supplementary file 8). The downregulated DEGs at 10 dpi were not significantly enriched. However, most of them were activated in the “ubiquitin-mediated proteolysis”, “synaptic vesicle cycle”, “collecting duct acid secretion” and “oxidative phosphorylation” (Fig. 3B). Together, the results of the functional enrichment analysis suggest that most of the upregulated DEGs were related to immune and inflammatory responses, and the downregulated genes to the ubiquitin system and oxidative phosphorylation.

### 3.4 Many genes showed similar expression patterns in the infected animals from 7 to 14 dpi

**Fig. 4:**
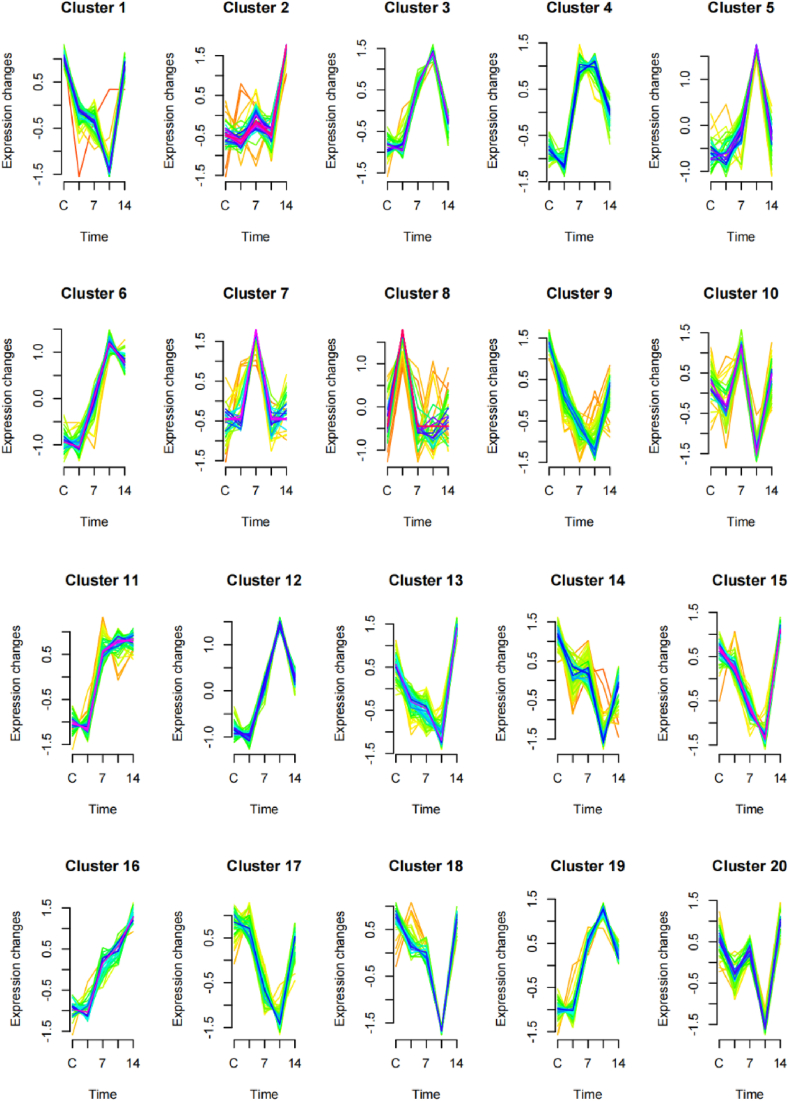
**Clusters of the DEGs based on their expression pattern over time.** Green and yellow colored lines correspond to genes with low membership value; purple and blue colored lines correspond to genes with high membership value. The x-axis is the time point of sample collection. C, control. (For interpretation of the references to color in this figure legend, the reader is referred to the Web version of this article.)

To gain an overview of the expression patterns of the DEGs, we clustered them using the Mfuzz package. The 857 DEGs were clustered into 20 clusters (Fig. 4) (supplementary file 9). The genes grouped in clusters 4, 6, and 11 showed the highest expression in the infected groups from 7 to 14 dpi (Fig. 4). Then, we performed GO/KEGG enrichment analysis for the genes from these clusters. The results showed that no GO terms or KEGG pathways were significantly enriched. The expression level of the DEGs from clusters 9 and 14 were decreased after infection (Fig. 4). The expression level of genes from the other clusters was increased at some time points and decreased at others (Fig. 4).

### 3.5 qPCR-based analysis of the DEGs

**Fig. 5:**
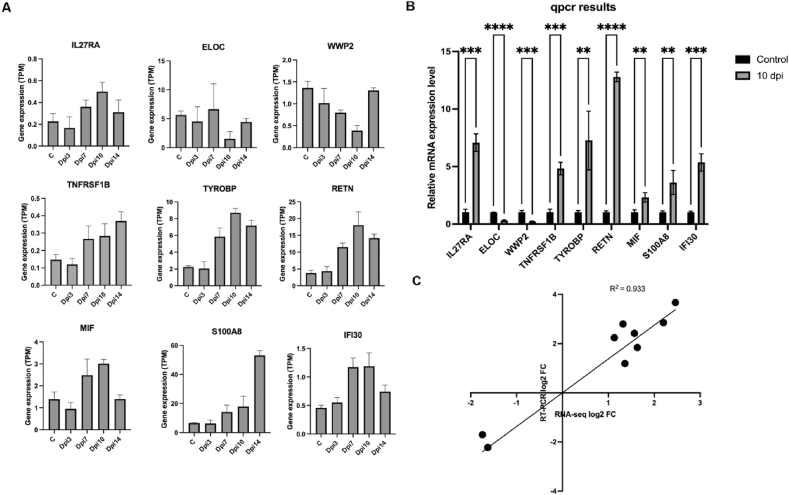
**Relative expression of the DEGs selected for the Illumina sequencing expression pattern validation**. (A) Illumina sequencing expression patterns of the nine genes selected for qPCR. (B) qPCR result for 10 Dpi compared to control *∗P* < 0.05, ∗∗*P* < 0 0.01, ∗∗∗*P* < 0.001, ∗∗∗∗*P* < 0.0001. ELOC and WWP2 were downregulated at Dpi 10. IL27RA, TNFRSF1B, TYROBP, RETN, S100A8, IFI30, and MIF were upregulated at Dpi 10. (C) Comparison of log2 fold change derived from RNA-seq with log2 fold change derived from qPCR. R^2^ corresponds to the coefficient of correlation.

For the validation of the RNA-seq results, we randomly selected nine DEGs (Fig. 5A) and detected their expression by qPCR (Fig. 5B) taking 10 dpi as a reference. The qPCR and NGS were performed on the same samples. As shown in Fig. 5C, the results obtained from qPCR were consistent with the expression pattern of the RNA-seq suggesting the reliability of the data. The tumor necrosis factor receptor superfamily member 1B (*TNFRSF1B*), macrophage migration inhibitory factor (*MIF*), gamma-interferon-inducible lysosomal thiol reductase (*IFI30*), interleukin-27 receptor subunit alpha (*IL2*7RA), *TYROBP*, *RETN*, S100A8 were up-regulated whereas transcription elongation factor B polypeptide 1(ELOC) and NEDD4-like E3 ubiquitin-protein ligase WWP2 (*WWP2*) were down-regulated (Fig. 5B).

## 4 Discussion

*M. fortis* is a non-susceptible host of *S. japonicum*. The mechanism of its resistance to schistosome infection may offer a route for the vaccine development or therapy. Although few *de novo* genomic and transcriptomic assemblages and analyses were carried out in liver samples, studies are still constrained by the lack of sufficient knowledge of the genomic resources of this rodent (H [11]). Here we examined the whole blood mRNA transcriptomic changes in *M. fortis* throughout the early stage of infection using the Illumina sequencing technology.

After quality control, we obtained 5310 sequences as unigenes and successfully annotated 4636 sequences. We observed that the number of genes responding to the invading schistosome was much higher at 10 dpi than at other time points post-infection. Our results are consistent with previous RNA-seq data from the liver samples suggesting that the intense immune response against the invading schistosomes occurs at 10 dpi (H. [11,16]). Schistosome interact with the final host at every stage of infection via different excretory/secretory (ES) proteins. These ES proteins regulate the host immune response [24]. The two developmental stages of schistosome in the final host are schistosomula (juvenile worm) and adult worm. In this study, we observed that the immune response was more intense at 10 dpi. This indicates that the ES proteins secreted by the invading schistosomula of 10 dpi might be more immunogenic than those secreted at the other time points. Further study of the immunogenicity of the ES proteins secreted by the juvenile worm in *M. fortis* will be useful for the identification of candidate antigens in the context of vaccine development.

GO enrichment analysis showed that the DEGs from 3 to 7 dpi were not significantly enriched. Those from 10 dpi were enriched in 103 GO terms for the upregulated DEGs and 12 GO terms for the downregulated DEGs. We observed that most of the upregulated DEGs were involved in leucocyte activation and migration. Leucocytes are essential for fighting invading pathogens. Their migration from the blood to the infected tissue manifests inflammation [25].

By mapping the DEGs against the KEGG database for functional enrichment analysis we observed that the upregulated genes were significantly enriched in several immune pathways at 10 dpi including TNF and NF-kappa B signaling pathways, Th1, Th2, Th17 cell differentiations, and cytokine-cytokine receptor interaction. These results were similar to previous studies [16] (H [11]). TNF signaling pathway is essential for many biological processes such as immune cell differentiation, regulation of immune response, and induction of inflammation [26]. This pathway triggers NF-kB-dependant response to exert its biological function [27]. TNF can also interact with TNF receptor 2 (*TNFR2* also known as *TNFRSF1B*) on T cells to promote their differentiation toward the Th1 and Th17 cells [28]. Interestingly, we found that the *TNFRSF1B* was significantly upregulated in the infected *M. fortis* at 10 dpi. The role of Th1 in immunity has been well studied in schistosomiasis but the mechanism of Th17 induction and its immunity is not well known [29]. However, there is an evidence that the Th17 contributes to the extracellular pathogen clearance [30]. In our study, 10 up-regulated genes including the interleukin-1 beta (IL1B), H-2 class II histocompatibility antigen (*MHC2*), protein c-Fos (*FOS*), tyrosine-protein kinase JAK3 (*JAK3*), hypothetical protein A6R68_17782 (*TRBV*), NF-kappa-B inhibitor alpha (*NFKBIA*), tyrosine-protein kinase ZAP-70 (*ZAP70*), T-cell surface glycoprotein CD3 delta chain (*CD3D*), retinoic acid receptor alpha (*RARA*) and *IL2*7RA were activated in the Th17 differentiation pathway. All the above evidence suggests that the TNF signaling pathway and Th17-mediated immunity might play a critical role in the mechanism of *M. fortis* defense against *S. japonicum*.

Subsequent analysis of the DEGs revealed that the *IF27L2B* was significantly upregulated in the infected animals from 3 to 14 dpi. This indicates that the *IF27L2B* may be associated with the mechanism of *M. fortis* resistance. In addition, we observed that the *RETN*, *PGRP*, *IFI35*, *TYROBP*, *S100A11*, *CD162* and *CD88* were significantly upregulated from 7 to 14 dpi. Whereas, the*S100A8*, *CYBA*, and *LBP* were significantly upregulated from 10 to 14 dpi. Although the roles of S100A8 and *S100A11* have not yet been elucidated in schistosomiasis, the *S100A* proteins are known as effector molecules that can block the development of several invading pathogens such as *Salmonella typhimurium* and *Staphylococcus aureus* [31]. In addition, they can interact with different pattern recognition receptors (PRRs) to promote the upregulation of proinflammatory genes via the NF-kB signaling pathway [32]. *CYBA* is involved in macrophage-mediated immunity. Its upregulation increases reactive oxygen species (ROS) generation, which promotes M1 macrophage polarization and inflammatory response [33].

Inflammatory responses are essential for clearing the invading schistosomula during the early stage of infection [34,35]. As mentioned above, the expression level of several proinflammatory genes including *TYROBP*, S100A8, *S100A11*, *LBP* and *CD88* were increased significantly after infection. The *CD88* gene promotes the recruitment and activation of myeloid cells during the infection [36]. *RETN* can also contribute to the invading parasite clearance by promoting the recruitment of inflammatory monocytes [37].

Our results complement the previous studies’ findings [16] (H [11]). We identified several potential candidate genes not previously associated with the mechanism of *M. fortis* resistance. Although we were able to explore the immune defense mechanism of *M. fortis* from the perspective of the whole blood transcriptome, our study has some limitations. The number of sequences identified as unigene was low, which affected the statistical power of the DEG analysis. Further studies involving the peripheral blood mononuclear cells, lung, liver, spleen lymph nodes, and thymus could identify more candidate genes and provide more detailed information about the *M. fortis* immune system.

## 5 Conclusion

In this study, we comprehensibly described the blood transcriptome profile of *M. fortis* for a better understanding of the mechanism of its resistance to *Schistosoma*. We identified 54 DEGs at 3 dpi, 228 at 7 dpi, 706 at 10 dpi, and 120 at 14 dpi. GO and KEGG enrichment analysis demonstrated that most of the upregulated genes were involved in the inflammatory and immune responses. The downregulated genes were mainly related to the ubiquitin system and oxidative phosphorylation. Based on our RNA-seq results, we suggest that the following processes might contribute to the worm killing in *M. fortis*: (1) The S100 proteins promote an inflammatory response or chelate the metal nutrients of the worms and block their development and (2) the *CD88* and *RETN* promote the recruitment of leucocytes which mediate inflammation and phagocytosis, resulting in the invading schistosomula destruction.

## Funding

This work was supported by grants from the 10.13039/501100001809National Natural Science Foundation of China (nos. 82102428 to S.H., nos. 82072306 and 32370197 to X.W.), the 10.13039/501100004735Natural Science Foundation of Hunan Province (nos. 2022JJ40663 to S.H.), the 10.13039/501100012166National Key Research and Development Program of China (No. 2022YFC2304001 to X.W.) and the Innovation Platform Open Foundation of 10.13039/100009377Education Department of Hunan Province, China (nos. 19K077 to Y.L.), Science and Technology Major Project of Changsha City (No. kh2301027 to Z.Z.).

## Availability of data and materials

The [RNA-Sequencing] data reported in this manuscript have been deposited at [NCBI's Sequence Read Archive (https://www.ncbi.nlm.nih.gov/sra)] with accession number [PRJNA1019071].

## Ethics approval and consent to participate

We obtained the ethical approval of the ethics committee of Central South University under license number 2021-KT25 before starting the study.

## Consent for publication

The manuscript does not include any details, images, or video relating to a person or human.

## CRediT authorship contribution statement

**Nouhoum Dibo:** Writing – review & editing, Writing – original draft, Software, Methodology, Investigation, Formal analysis, Conceptualization. **Zhijun Zhou:** Writing – review & editing, Conceptualization. **Xianshu Liu:** Methodology, Formal analysis. **Zhuolin Li:** Software, Methodology, Formal analysis. **Shukun Zhong:** Methodology, Formal analysis. **Yan Liu:** Writing – review & editing, Funding acquisition. **Juan Duan:** Writing – review & editing, Methodology. **Meng Xia:** Writing – review & editing, Formal analysis. **Zhenrong Ma:** Methodology, Formal analysis. **Xiang Wu:** Writing – review & editing, Supervision, Funding acquisition, Conceptualization. **Shuaiqin Huang:** Writing – review & editing, Writing – original draft, Funding acquisition, Formal analysis, Conceptualization.

## Declaration of competing interest

We wish to confirm that there are no known conflicts of interest associated with this publication and there has been no significant financial support for this work that could have influenced its outcome.
