# Supplementary material for: Time-course whole blood transcriptome profiling provides new insights into Microtus fortis natural resistance mechanism to Schistosoma japonicum
Source: Heliyon. 2024 Sep 26;10(19):e38067. doi: 10.1016/j.heliyon.2024.e38067 (PMC11471165; doi:10.1016/j.heliyon.2024.e38067)

**Time-course whole blood transcriptome profiling provides new insights into *Microtus fortis* natural resistance mechanism to *Schistosoma japonicum***

**Supplementary figure 1**


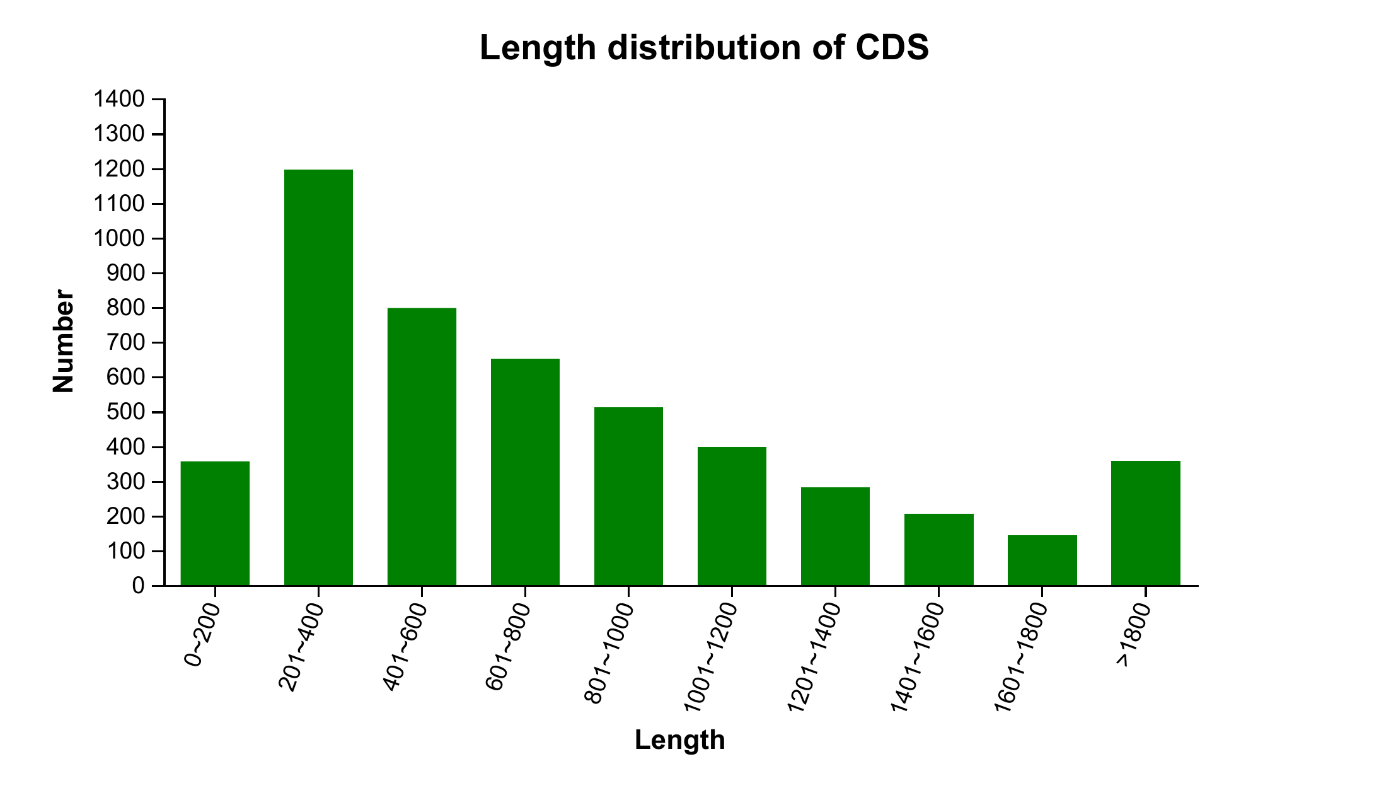

Supplement: Multimedia component 10 [file mmc10.docx]
