# Supplementary material for: Time-course whole blood transcriptome profiling provides new insights into Microtus fortis natural resistance mechanism to Schistosoma japonicum
Source: Heliyon. 2024 Sep 26;10(19):e38067. doi: 10.1016/j.heliyon.2024.e38067 (PMC11471165; doi:10.1016/j.heliyon.2024.e38067)
Supplement: Multimedia component 11 [file mmc11.docx]

**Time-course whole blood transcriptome profiling provides new insights into Microtus fortis natural resistance mechanism to Schistosoma japonicum**

**Supplementary table1** List of the primer used for qPCR

| **Gene** | **Forward/Reverse** | **Primer sequence (5'->3')** | **NCBI Reference Sequence** |
| --- | --- | --- | --- |
| Mif | F | GCAGGATGCCAAGAAGTCCT | XM_050143207.1 |
|  | R | CAGTGACAGGGGCAACTTGA |  |
| S100A8 | F | CTCTGTCTCAGGGACATCGG | XM_050118678.1 |
|  | R | GAGGGCATGGTTGTTCCCTT |  |
| Retn | F | CGAGAGGGAACAACGTGTCA | XM_050127400.1 |
|  | R | GCTTTCAGGGATCCACACCA |  |
| Ifi30 | F | CGAGATCTGTTCCCGACCTG | XM_050127588.1 |
|  | R | TAGCAACGCAGCCTCCTTTT |  |
| Tyrobp | F | CACTTGGTTGGACGGGACA | XM_050119231.1 |
|  | R | GAACAGCTGCACTCTCCCAC |  |
| IL27RA | F | GCCTTCGGCTTCACCTACCA | XM_050120661.1 |
|  | R | TGCCCAGAATTGCTGTTGGC |  |
| ELOC | F | GAAAGGGCGAGAACGACAACG | XM_050142067.1 |
|  | R | TGACGTACATGGCGTCAGGG |  |
| WWP2 | F | ATATGATGGACCACGCCCTCC | XM_050144083.1 |
|  | R | ACTTTCAGGGTGAGCTGGGAC |  |
| TNFRSF1B | F | TTGCCGGTCAAGTGAGGGTT | XM_050153318.1 |
|  | R | ACTGCCCCATGGAACACCAA |  |
| Actb | F | GCAGGAGTACGATGAGTCCG | XM_050163204.1 |
|  | R | AAACGCAGCTCAGTCACAGT |  |

**Supplementary table 2** GO enrichment analysis results for 3 dpi

| **Number of DEG** | **Up/down** | **GO ID** | **Description** | **P-value corrected** |
| --- | --- | --- | --- | --- |
| 2 | Up | GO:0042698 | ovulation cycle | 1 |
| 4 | Up | GO:0048609 | multicellular organismal reproductive process | 1 |
| 3 | Up | GO:0071456 | cellular response to hypoxia | 1 |
| 3 | Up | GO:0036294 | cellular response to decreased oxygen levels | 1 |
| 3 | Up | GO:0071453 | cellular response to oxygen levels | 1 |
| 1 | Down | GO:1901585 | regulation of acid-sensing ion channel activity | 1 |
| 1 | Down | GO:0032515 | negative regulation of phosphoprotein phosphatase activity | 1 |
| 1 | Down | GO:0035308 | negative regulation of protein dephosphorylation | 1 |
| 1 | Down | GO:0044829 | positive regulation by host of viral genome replication | 1 |
| 1 | Down | GO:0045747 | positive regulation of Notch signalling pathway | 1 |

**Supplementary Table 3** GO enrichment analysis results for 7 dpi

| Number of gene | Up/down | GO ID | Description | P-value corrected |
| --- | --- | --- | --- | --- |
| 4 | Up | GO:0070232 | regulation of T cell apoptotic process | 0.136941028 |
| 4 | Up | GO:0070228 | regulation of lymphocyte apoptotic process | 0.632434498 |
| 7 | Up | GO:0044282 | small molecule catabolic process | 0.632434498 |
| 16 | Up | GO:0002376 | immune system process | 0.632434498 |
| 4 | Up | GO:0071675 | regulation of mononuclear cell migration | 0.906572923 |
| 3 | Down | GO:0040020 | regulation of meiotic nuclear division | 0.90914145 |
| 5 | Down | GO:0006513 | protein monoubiquitination | 0.90914145 |
| 3 | Down | GO:0051445 | regulation of meiotic cell cycle | 0.90914145 |
| 10 | Down | GO:0055080 | cation homeostasis | 0.90914145 |
| 3 | Down | GO:0033522 | histone H2A ubiquitination | 0.90914145 |
